# α-Hederin Alleviates Endoplasmic Reticulum Stress by Upregulating TRIM38 Expression, Thereby Inhibiting Hepatic Stellate Cell Activation and Liver Fibrosis

**DOI:** 10.3390/biomedicines14040829

**Published:** 2026-04-05

**Authors:** Wei Xu, Yang Yang, Fuqiang Li, Can Li, Gaojun Tang, Baofang Zhang, Mingliang Cheng

**Affiliations:** 1 Department of Gastroenterology, The Affiliated Hospital of Guizhou Medical University, Guiyang 550008, China; 2 Department of Nephrology, The First People’s Hospital of Guiyang, Guiyang 550008, China; 3 Department of Infectious Diseases, The Affiliated Hospital of Guizhou Medical University, Guiyang 550008, China

**Keywords:** liver fibrosis, LX-2, α-hederin, TRIM38, endoplasmic reticulum stress

## Abstract

**Objectives**: This study aims to investigate the potential molecular mechanisms by which α-hederin modulates HSC activation to alleviate liver fibrosis. **Methods**: An in vitro model of liver fibrosis was established by inducing LX-2 cells with TGF-β1. These cells were then treated with α-hederin (10 μg/mL) before undergoing phenotypic analysis and molecular-level detection. A mouse model of liver fibrosis induced by CCl_4_ was established in vivo to further evaluate the expression levels of fibrosis markers, including TRIM38. **Results**: In TGF-β1-induced liver fibrosis in LX-2 cells, α-hederin treatment significantly inhibited HSCs activation, as evidenced by down-regulation of α-SMA and suppressed proliferation capacity. At the same time, α-hederin significantly reduced the levels of COL1A1, COL3A1, fibronectin, and MMP-2. Transcriptome sequencing analysis revealed that α-hederin treatment significantly upregulated TRIM38 expression. Differentially expressed genes (DEGs) were significantly enriched in endoplasmic reticulum stress-related pathways. TRIM38 up-regulation inhibits HSC activation and proliferation, reducing the expression of ERS marker proteins (GRP78, p-PERK, and CHOP); Co-IP experiments further confirmed that TRIM38 and GRP78 interact directly. Further rescue experiments demonstrated that TRIM38 knockdown significantly attenuated the inhibitory effects of α-hederin on these processes. In a CCl_4_-induced mouse model of liver fibrosis, α-hederin (4 mg/kg) significantly reduced the liver index and serum ALT and AST levels, improved histopathological damage to the liver, upregulated TRIM38 expression in liver tissue, and inhibited the endoplasmic reticulum stress response (ERS). **Conclusions**: α-hederin exerts its anti-fibrotic effect by upregulating TRIM38, thereby alleviating endoplasmic reticulum stress and ultimately inhibiting the activation and proliferation of HSCs.

## 1. Introduction

Hepatic fibrosis (HF) results from dysregulated repair processes following hepatocyte injury caused by various etiologies, including viral hepatitis, alcoholic fatty liver disease, non-alcoholic fatty liver disease, drug-induced liver injury, cholestatic liver disease, and autoimmune liver disease. Its primary pathological feature involves increased synthesis and reduced degradation of the extracellular matrix (ECM), leading to excessive deposition that disrupts the liver’s physiological architecture [1]. Without timely intervention, fibrosis may progress to cirrhosis or even hepatocellular carcinoma, making it one of the leading causes of liver disease-related mortality worldwide [2,3]. At present, most candidates discontinued treatment due to insufficient efficacy or adverse reactions [4]. No “specific” anti-fibrotic drugs for liver fibrosis have been approved for market release to date. Clinical management remains centered on addressing underlying causes (antiviral therapy, alcohol cessation, weight loss/metabolic interventions), while anti-fibrotic drug development continues to explore multiple targets and pathways in parallel [5,6,7].

Hepatic stellate cell (HSCs) activation is a central driver of liver fibrosis. Quiescent HSCs store retinol and secrete minimal matrix metalloproteinases, but upon stimulation by oxidative stress or inflammatory mediators, they transdifferentiate into myofibroblast-like cells characterized by proliferation, contractility, and excessive ECM synthesis [7,8]. This process involves epigenetic reprogramming, signaling network remodeling, and subcellular organelle restructuring [9,10,11,12]. Notably, endoplasmic reticulum stress (ERS) has emerged as a key contributor to HSC activation [12,13]. Persistent ERS activates three classical pathways—IRE1α/XBP1s, PERK/eIF2α/ATF4, and ATF6—which enhance protein folding, promote lipid synthesis, and upregulate pro-fibrotic genes such as TGF-β1 and CTGF [14,15,16]. Furthermore, ERS-induced autophagy degrades lipid droplets to fuel the highly secretory phenotype of activated HSCs, thereby accelerating fibrosis progression [7]. Targeting ERS and its downstream effectors thus represents a promising anti-fibrotic strategy.

TRIM38 is a TRIM family E3 ubiquitin ligase containing the conserved RBCC motif, with established roles in innate immunity, tumorigenesis, and inflammation [17,18]. In myocardial hypoxia/reperfusion models, TRIM38 over-expression reduces oxidative stress and inhibits TAK1/NF-κB signaling to mitigate cell death [19]. In NASH models, TRIM38 deficiency exacerbates lipid metabolism disorders, inflammation (NF-κB/IL-17/TNF-α), and ECM deposition, whereas over-expression alleviates these pathologies [20]. Regarding fibrosis, TRIM38 inhibits cardiac fibroblast proliferation and secretory activity [21] and suppresses hepatocyte steatosis and fibrosis by targeting TAB2 to block TAK1/MAPK signaling [22]. However, whether TRIM38 directly regulates HSC activation—the core driver of liver fibrosis—remains unknown. Given that oxidative stress and ERS synergistically promote HSC activation, we hypothesize that TRIM38 may influence HSC fate by modulating ERS thresholds, a mechanism requiring experimental clarification.

Natural products represent a significant source of anti-fibrotic compounds, with triterpenoid saponins attracting particular attention for their multi-target regulatory properties. α-Hederin, a monosaccharide-linked pentacyclic triterpenoid saponin isolated from Hedera helix or Nigella sativa [23], exhibits broad pharmacological activities including anti-tumor, antibacterial, and anti-inflammatory effects. Its anti-tumor mechanisms involve inducing apoptosis and cell cycle arrest, reducing ATP production, and inhibiting autophagy, proliferation, and metastasis [24,25,26]. Notably, α-hederin inhibits TGF-β/SMAD2 and SREBF1/FASN signaling, thereby reducing lipid synthesis and ECM deposition [27]. Given that TGF-β/SMAD is a classic driver of fibrosis [28], α-hederin may have therapeutic potential in liver fibrosis. Additionally, α-hederin regulates ERS signaling to induce tumor cell apoptosis [25], suggesting a possible mechanism in fibrotic contexts. However, whether α-hederin definitively ameliorates liver fibrosis and whether this depends on ERS regulation remains unclear due to limited systematic investigation. This study established an in vitro model of hepatic stellate cell activation and a CCl_4_-induced mouse model of liver fibrosis to investigate whether α-hederin suppresses endoplasmic reticulum stress by upregulating TRIM38 expression, thereby influencing the activation and proliferation of HSCs.

## 2. Materials and Methods

### 2.1. Pharmaceuticals and Reagents

α-hederin (Purity ≥ 98%, Shanghai MCE, Shanghai, China); TGF-β1 recombinant protein (MCE, HY-P7118, Monmouth Junction, NJ, USA); CCK-8 (Dojindo, CK04, Kumamoto, Japan); EdU Kit (RiboBio, C10310, Guangzhou, China); TRIzol (Invitrogen, Carlsbad, CA, USA); Reverse Transcription Reagent (Takara, RR047A, Shiga, Japan); SYBR Green (Roche, 04707516001, Basel, Switzerland); Antibodies: α-SMA (Protein tech, 14395-1-AP, Rosemont, IL, USA), TRIM38 (Protein tech, 13405-1-AP), PERK (Protein tech, 68482-1-Ig), p-PERK (82534-1-RR, 82534-1-RR), GRP78 (ABclonal, A23453, Woburn, MA, USA), CHOP (ABclonal, A21902), β-actin (Protein tech, 66009-1-Ig), β-tubulin (Protein tech, 10094-1-AP). COL1A1 Kit (Jiangsu Enzyme-Linked Bio, Yancheng, China); COL3A1 Kit (Jiangsu Enzyme-Linked Bio, China); Fibronectin Kit (Jiangsu Enzyme-Linked Bio, China); MMP-2 Kit (Jiangsu Enzyme-Linked Bio, China); ALT Kit (Nanjing Jiancheng, China, C009-2-1, Nanjing, China); AST Kit (Nanjing Jiancheng, China, C010-2-1); GRP78 (Proteintech, 11587-1-AP, Rosemont, IL, USA).

### 2.2. Cell Culture

The human hepatocyte stellate cell line LX-2 was purchased from Shanghai Saibai Kang Biotechnology Co., Ltd., Shanghai, China. The cells were cultured routinely in DMEM medium supplemented with 10% fetal bovine serum (FBS) and 1% penicillin-streptomycin at 37 °C in a 5% CO_2_ incubator.

### 2.3. CCK-8 Assay for Cell Viability

Seed LX-2 cells at a density of 5 × 10^3^ cells per well in a 96-well plate. Prepare a stock solution of a-hederin by dissolving it in DMSO; dilute to the working concentration with culture medium when using. After attachment, replace the medium with DMSO (0.1%) and α-hederin at different concentrations (5, 10, 15, 20, 25, 30 μg/mL) for 24 h. Add CCK-8 reagent and incubate for 2 h. Measure the absorbance at 450 nm and calculate the cell survival rate.

### 2.4. EdU Staining

Seed LX-2 cells at a density of 2 × 10^6^ cells per well in a 6-well plate. After cells adhere, pre-treat with TGF-β1 (10 ng/mL) for 2 h, followed by α-hederin (10 μg/mL) for 24 h. After treatment, add 10 μM EdU working solution to the medium and incubate at 37 °C in the dark for 2 h. Observe EdU-positive cells using an inverted fluorescence microscope (Olympus, Tokyo, Japan). Detect proliferating cells via dual staining with EdU and DAPI.

### 2.5. Immunofluorescence Staining

Cells were fixed with 4% paraformaldehyde, permeabilized with 0.3% Triton X-100 for 10 min, and blocked with 5% BSA at room temperature for 1 h. Primary antibody was added and incubated overnight at 4 °C. After washing with PBS, Alexa Fluor 488- or 594-labeled secondary antibody was incubated at room temperature in the dark for 1 h. Nuclei were counterstained with DAPI. Images were acquired under a fluorescence microscope (Olympus, Tokyo, Japan). Five representative fields of view were randomly selected from each sample. Quantitative analysis was performed using ImageJ 2.15.1 software; following background subtraction and the application of a standardized threshold, the mean fluorescence intensity was measured. The mean of the data from the multiple fields of view for each sample was taken as the representative value for that biological replicate; the final statistical analysis was based on the number of biological replicates rather than technical replicates.

### 2.6. Transcriptome Sequencing Analysis

Transcriptomic analysis was performed on the following two groups of LX-2 cells: (1) TGF-β1 model group: treated with 10 ng/mL TGF-β1 alone for 24 h; (2) α-hederin intervention group: pretreated with 10 ng/mL TGF-β1 for 2 h, followed by co-culture with 10 μg/mL α-hederin for 24 h. Total cellular RNA was extracted using TRIzol reagent. After quality control, strand-specific library preparation and paired-end sequencing (RNA-seq) were performed on the Illumina NovaSeq platform. Raw data underwent quality control, alignment (reference genome GRCh38), and gene expression quantification. Differential expression analysis was performed using DESeq2 with a screening criterion of |log2FoldChange| > 1 and FDR < 0.05. The identified DEGs were further analyzed using GO functional analysis and KEGG pathway enrichment analysis.

### 2.7. Cell Transfection and Stable Cell Line Construction

TRIM38 over-expression and knockdown lentiviral vectors were constructed and synthesized by Shanghai Heyuan Biotechnology Co., Ltd., Shanghai, China. To establish stable TRIM38-expressing cell models, LX-2 cells were infected with lentiviruses carrying either the TRIM38 over-expression sequence (OE-TRIM38) or the short hairpin RNA knockdown sequence (sh-TRIM38). Cells transfected with empty vector viruses (OE-NC or sh-NC) served as controls. Twenty-four hours prior to infection, LX-2 cells were seeded at 2 × 10^6^ cells/well in 6-well plates. Upon reaching 60–70% confluence, corresponding lentiviral solutions were added at a multiplicity of infection (MOI = 20), supplemented with 6 μg/mL Polybrene to enhance infection efficiency. Forty-eight hours post-infection, GFP expression was assessed under fluorescence microscopy to evaluate transfection efficiency. Subsequently, continuous selection was performed using complete medium supplemented with 2 μg/mL puromycin to establish a stably transfected cell line. Finally, TRIM38 expression was validated at both the mRNA and protein levels via RT-qPCR and Western blot, respectively, to confirm the successful construction of the stably transfected cell line.

### 2.8. Co-Immunoprecipitation (CoIP)

Cells were harvested and lysed directly in the culture flask using mild RIPA buffer for 30 min. Meanwhile, 50 µL of Dynabeads Protein G (Life Technologies) was incubated with 3 µg of antibody at room temperature for 1 h. The protein lysate was then mixed with the magnet bead–antibody complex and incubated overnight at 4 °C. The magnet beads were washed three times with lysis buffer. The bound protein and a 10% Blank Control were detected by Western blotting.

### 2.9. Laboratory Animal

SPF-grade male C57BL/6 mice (weighing 18–22 g) were purchased from Beijing Sbeifu Biotechnology Co., Ltd., Beijing, China. Mice aged 6–8 weeks were housed in accordance with standard animal facility protocols in a Controlled environment with a constant temperature (22 ± 2 °C) and humidity (50 ± 10%), under a 12 h light/12 h dark cycle, with ad libitum access to food and water. They were acclimatized for one week prior to the experiment. A mouse model of liver fibrosis was established by intraperitoneal injection of a mixture of olive oil and CCl_4_. Method: CCl_4_ was dissolved in olive oil at a concentration of 20% (*v*/*v*) and administered at a dose of 0.6 mL/100 g twice weekly for 4 weeks. Concurrently with model induction, the mice were randomly divided into six groups (eight mice per group): a normal Control group (equal volume of olive oil), a model group (CCl_4_ + equal volume of saline), a low-dose α-hederin group (2 mg/kg α-hederin), a medium-dose α-hederin group (4 mg/kg α-hederin), a high-dose α-hederin group (8 mg/kg α-hederin), and a positive control group (2 mg/kg Bupleurum saponin D). The treatment groups received intraperitoneal injections of the corresponding drugs once daily for 4 consecutive weeks. Following the final dose, the mice were fasted (but not deprived of water) for 12 h. They were then anesthetized with 3% isoflurane, and blood was collected from the retro-orbital venous plexus before euthanasia. The blood was left to stand at room temperature for 30 min, then centrifuged at 3000 rpm at 4 °C for 10 min. The supernatant serum was collected and stored at −80 °C for later use. The liver was rapidly excised, pre-cooled, and washed with PBS to remove residual blood. The left hepatic lobe was excised and fixed in 4% paraformaldehyde for 24 h, followed by routine dehydration and paraffin embedding; the remaining liver tissue was rapidly frozen in liquid nitrogen and transferred to a −80 °C freezer for storage, to be used for Western blot and biochemical parameter analysis.

### 2.10. RNA Extraction and RT-qPCR

Total RNA was extracted from cells using TRIzol reagent. Reverse transcription was performed with the RevertAid First-Strand cDNA Synthesis Kit. PCR reactions were conducted using a real-time quantitative PCR system (Applied Biosystems, Foster City, California, USA), with β-actin as the internal control. Relative gene expression levels were calculated using the 2^−ΔΔCt^ method. Primer sequences for each gene are listed in Table 1.

### 2.11. Western Blot Analysis

The cells/liver tissue were collected, and pre-chilled lysis buffer containing protease inhibitors was added. These were ice-incubated for 30 min. Then, they were centrifuged at 13,000 RPM at 4 °C for 30 min. The supernatant was aspirated. After quantifying the proteins, the loading buffer was added and boiled for 5 min. After electrophoresis, the membrane was transferred. It was blocked with skim milk, then washed with PBST. The primary antibody (1:1000) was diluted and incubated overnight at 4 °C. The next day, the bands were washed three times with cold PBS, incubated with the secondary antibody for one hour, and imaged using a chemiluminescent reader. Total cellular proteins were extracted and separated by SDS-PAGE. Then, they were transferred to membranes, blocked, and incubated with primary and secondary antibodies. Protein bands were visualized with ECL reagent; molecular weights were estimated using PageRuler™ Prestained Protein Ladder (Thermo Fisher, 26616, Waltham, MA, USA). The experimental design comprised three independent biological replicates, each consisting of three technical replicates run on a single gel; quantitative analysis was performed using ImageJ software, whereby the integrated optical density values of the bands were measured after background subtraction, and differences in loading amounts were corrected using a reference protein to calculate the relative expression levels of the target protein; statistical analysis was based on the mean values of the three independent biological replicates, rather than the technical replicate data, in order to accurately reflect biological variation.

### 2.12. Enzyme-Linked Immunosorbent Assay (ELISA)

Collect cell and mouse serum samples. Following the kit instructions strictly, sequentially measure the levels of COL1A1, COL3A1, fibronectin, and MMP-2 in LX-2 cells, as well as the ALT and AST levels in mouse serum.

### 2.13. HE Staining

Prepare paraffin sections and perform routine dewaxing to water. Immerse them in a hematoxylin stain solution for 5 min, then rinse under running water to remove excess dye. Fix in 1% hydrochloric acid in ethanol, then rinse under running water. Immerse them in an eosin stain solution for 3 min. Dehydrate them using a gradient of ethanol, clear with xylene, and mount with neutral resin. Observe the morphology, arrangement, degeneration, necrosis, and inflammatory cell infiltration of hepatocytes under a light microscope.

### 2.14. Masson’s Stain

Paraffin sections were routinely deparaffinised in water, and the following staining procedure was followed as per the kit instructions: Weigert’s iron-hematoxylin stain for nuclei (5 min); leucochrome acid–magenta stain for cytoplasm (5 min); 1% phosphomolybdic acid for differentiation (3 min); aniline blue stain for collagen fibers (5 min); and 1% glacial acetic acid for differentiation (1 min). Following dehydration, clearing, and mounting, collagen fiber deposition was observed under a light microscope (collagen appears blue, cytoplasm appears red, and nuclei appear blue-brown). Five random fields of view (×200) from each section were selected, and ImageJ 2.15.1 software was used to analyze the percentage of collagen area.

### 2.15. Immunohistochemistry (IHC)

Paraffin sections were routinely deparaffinised in water, followed by antigen retrieval in citrate buffer (pH 6.0) using a microwave for 15 min. Endogenous peroxidase was blocked with 3% H_2_O_2_ at room temperature for 10 min, followed by blocking with 5% BSA for 30 min. Rabbit anti-α-SMA primary antibody (1:200) was added dropwise and incubated overnight at 4 °C; the following day, HRP-labeled goat anti-rabbit secondary antibody (1:500) was added dropwise and incubated for 30 min at room temperature, followed by DAB color development and counterstaining of nuclei with haematoxylin. The sections were dehydrated, mounted with clear mountant, and examined under a light microscope; the percentage of positive staining area and mean optical density values were analyzed using ImageJ 2.15.1 software.

### 2.16. Statistical Analysis

All experimental data are expressed as mean ± standard deviation (SD). Statistical analysis was performed using GraphPad Prism 9.0 software. Comparisons between two groups were conducted using the independent samples *t*-test, while comparisons among multiple groups were performed using one-way analysis of variance (ANOVA). If the homogeneity of variance was satisfied, Tukey’s post hoc multiple comparisons were further applied. All experiments were independently replicated at least three times. Differences were considered statistically significant at *p* < 0.05.

## 3. Results

### 3.1. α-Hederin Inhibits LX-2 Cell Activation and Proliferation

**Figure 1:**
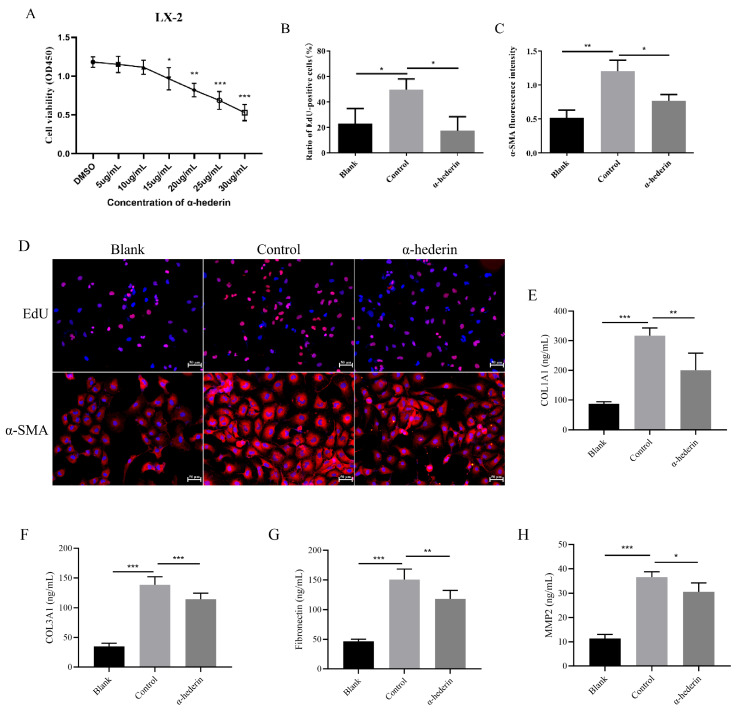
α-Hederin inhibits TGF-β1-induced activation of LX-2 cells. (**A**) CCK-8 assay of LX-2 cell viability after 24 h treatment with different concentrations of α-hederin. (**B**) EdU assay of TGF-β1-induced LX-2 cell proliferation. (**C**) Immunofluorescence detection of TGF-β1-induced α-SMA expression in LX-2 cells. (**D**) Representative images of immunofluorescence staining for EdU and α-SMA. (**E**,**F**) ELISA for collagen COL1A1 and COL3A1 levels. (**G**) ELISA for fibronectin levels. (**H**) ELISA for MMP-2 levels. *n* = 4–6. Scale bar: 50 μm. Groups: Blank, Control (TGF-β1), and α-hederin (TGF-β1 + α-hederin). * *p* < 0.05, ** *p* < 0.01, and *** *p* < 0.001.

To investigate the role of α-hederin in liver fibrosis, we conducted in vitro experiments using human-derived LX-2 hepatocyte stellate cells to observe its effects on LX-2 cell activation. First, we assessed the cytotoxic effects of various α-hederin concentrations on LX-2 cells. Based on preliminary observations of cell viability dynamics and changes in cell phenotype from the preliminary experiments, we selected 24 h as the duration of α-hederin treatment. CCK-8 assay results indicated that α-hederin inhibited cell proliferation at 15 μg/mL (Figure 1A). Consequently, we selected α-hederin (10 μg/mL) as the optimal treatment concentration for subsequent experiments. Following TGF-β1 stimulation of LX-2 cells, treatment with 10 μg/mL α-hederin was administered. EdU labeling assessed cell proliferation, while immunofluorescence evaluated expression of α-SMA, a marker of liver fibrosis. Results demonstrated reduced EdU-positive cell counts and decreased α-SMA fluorescence intensity in the α-hederin-treated group (Figure 1B–D). These results indicate that α-hederin suppresses HSC activation.

At the same time, we analyzed markers associated with extracellular matrix deposition; the results showed that α-hederin significantly reduced the levels of COL1A1, COL3A1, fibronectin, and MMP-2, thereby decreasing collagen deposition in HSCs (Figure 1E–H).

### 3.2. Upregulation of TRIM38 by α-Hederin Inhibits Endoplasmic Reticulum Stress and LX-2 Cell Activation

**Figure 2:**
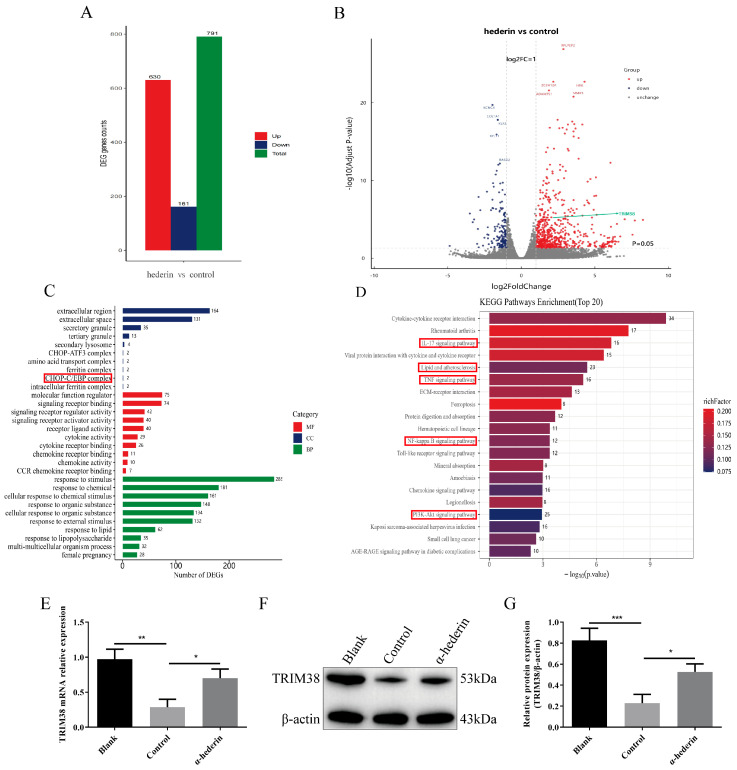
Effects of α-hederin on the LX-2 cell transcriptome and validation of TRIM38 expression. (**A**) Statistics of DEGs. (**B**) Volcano plot of DEGs: Red indicates up-regulation, blue indicates down-regulation. (**C**) GO enrichment analysis of DEGs (BP, CC, MF). (**D**) KEGG pathway enrichment analysis of DEGs. (**E**) RT-qPCR detection of TRIM38 mRNA expression. (**F**,**G**) Western blot detection of TRIM38 protein expression. *n* = 3–4. Groups: Blank, Control (TGF-β1), and α-hederin (TGF-β1 + α-hederin). * *p* < 0.05, ** *p* < 0.01, and *** *p* < 0.001.

In order to explore the molecular mechanism of α-hemin inhibiting LX-2 activation, we treated LX-2 cells activated by TGF-β1 with α-hemin and performed transcriptome sequencing to analyze changes in gene expression profiles (Supplementary Material Table S1). The results showed that compared with the TGF-β1 group, the α-hederin group identified 791 DEGs, including 630 upregulated genes and 161 down-regulated genes (Figure 2A). The volcano map intuitively displays the overall distribution of DEGs, revealing that the number of up-regulated genes is significantly higher than that of down-regulated genes, indicating that α-hederin mainly activates gene expression. Among them, TRIM38 is one of the significantly upregulated DEGs. (Figure 2B). GO functional enrichment analysis showed that DEGs were significantly enriched in cellular components such as the periphery, extracellular space, and plasma membrane, as well as in endoplasmic reticulum stress-related complexes (CHOP-C/EBP) (Figure 2C). These genes are mainly related to signal receptor binding and regulatory activity, and they are significantly enriched in response to stimuli and intercellular communication. KEGG pathway analysis further showed that the significantly enriched pathways included TNF signaling, NF-κB signaling, PI3K Akt signaling, IL-17 signaling, and lipid and atherosclerosis pathways (Figure 2D). These pathways are known to participate in and/or induce endoplasmic reticulum stress responses [29,30,31]. The transcriptome analysis systematically revealed extensive gene expression changes in LX-2 cells treated with α-hederin, particularly significant enrichment in pathways related to immune inflammation regulation and endoplasmic reticulum stress. This provides important clues for elucidating the potential anti-fibrotic mechanism of α-hederin in the liver.

GSEA results revealed that (Supplementary Material Table S2) pathways such as “Protein processing in the endoplasmic reticulum”, “Oxidative phosphorylation”, and “Cytokine-cytokine receptor interaction” were significantly enriched in the α-hederin-treated group across multiple databases, including GO, KEGG, REACTOME, and WIKIPATHWAY, which is highly consistent with the regulatory mechanism of the stress response mediated by TRIM38. Concurrently, biological processes such as “ubiquitin-protein ligase activity”, “unfolded protein binding”, and “endoplasmic reticulum stress response” were also significantly enriched. These results systematically elucidate the molecular network underlying α-hederin’s anti-fibrotic effects at the pathway level.

Based on transcriptome sequencing results indicating up-regulation of TRIM38 expression, we further validated TRIM38 expression at both mRNA and protein levels using RT-qPCR and Western blot. As shown in Figure 2E, compared to the Blank group, the TGF-β1-stimulated Control group exhibited significantly reduced *TRIM38* mRNA expression, which was markedly reversed following α-hederin intervention. This finding is consistent with sequencing results. Western blot analysis revealed that TRIM38 protein expression was significantly reduced in the Control group compared to the Blank group. However, α-hederin treatment effectively reversed this trend, leading to a significant rebound in TRIM38 protein expression (Figure 2F,G). These findings confirm that TRIM38 expression is suppressed during TGF-β1-induced LX-2 cell activation, and α-hederin significantly upregulates both transcriptional and translational levels of TRIM38.

**Figure 3:**
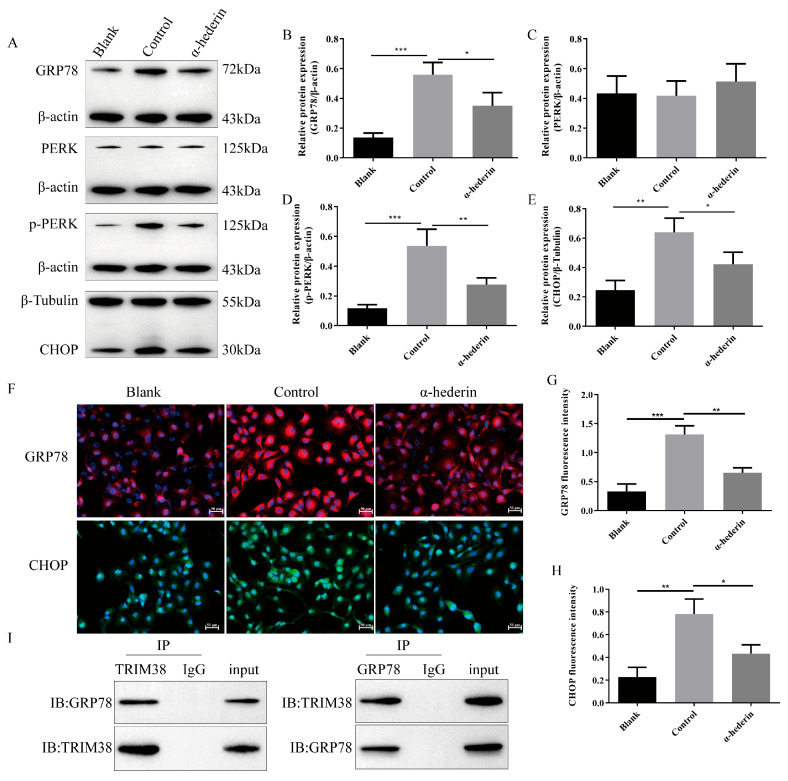
α-Hederin alleviates TGF-β1-induced endoplasmic reticulum stress in LX-2 cells by inhibiting the PERK/CHOP pathway. (**A**) Representative Western blot image. (**B**–**E**) Quantitative analysis of GRP78, PERK, p-PERK, and CHOP proteins. (**F**) Representative immunofluorescence images. (**G**,**H**) Immunofluorescence detection of GRP78 and CHOP expression. (**I**) Representative images of COIP. *n* = 3–4, Scale bar: 50 μm, Groups: Blank, Control (TGF-β1), α-hederin (TGF-β1 + α-hederin). * *p* < 0.05, ** *p* < 0.01, *** *p* < 0.001.

Transcriptome sequencing suggested that α-hederin function may be associated with endoplasmic reticulum stress. We further validated the PERK/CHOP pathway within this network. Western blot analysis (Figure 3A–E) revealed that compared to the Blank group, the TGF-β1-stimulated Control group exhibited significantly elevated expression of key ERS proteins GRP78, CHOP, and p-PERK, while total PERK levels remained unchanged, confirming successful ERS activation. Following α-hederin intervention, the expression of GRP78, CHOP, and p-PERK was significantly reduced compared to the Control group, suggesting that α-hederin effectively alleviates TGF-β1-induced ERS. This result was further confirmed at the cellular morphology level. Immunofluorescence analysis (Figure 3F–H) revealed markedly enhanced fluorescence intensity of GRP78 and CHOP in the Control group cells compared to the Blank group. Following α-hederin treatment, the fluorescence signals of both markers were significantly attenuated, clearly demonstrating the inhibitory effect of α-hederin on ERS. Collectively, these results demonstrate that α-hederin significantly suppresses TGF-β1-induced endoplasmic reticulum stress in LX-2 cells, potentially through down-regulating PERK/CHOP pathway activity. To investigate the molecular mechanism by which α-hederin regulates the ERS, we further validated the protein–protein interaction between TRIM38 and GRP78. Results from bidirectional immunoprecipitation (Co-IP) experiments (Figure 3I) showed that GRP78 protein was detectable in the TRIM38 antibody precipitate, whilst TRIM38 protein was also detectable in the GRP78 antibody precipitate; no specific binding was observed in the IgG Control group, confirming that TRIM38 and GRP78 interact directly in LX-2 cells. This finding suggests that α-hederin may regulate the activation of the GRP78-mediated PERK-CHOP pathway by enhancing the binding between TRIM38 and GRP78, thereby ultimately alleviating endoplasmic reticulum stress.

### 3.3. Effects of TRIM38 Gain-of-Function and Loss-of-Function on LX-2 Cell Activation and Endoplasmic Reticulum Stress

**Figure 4:**
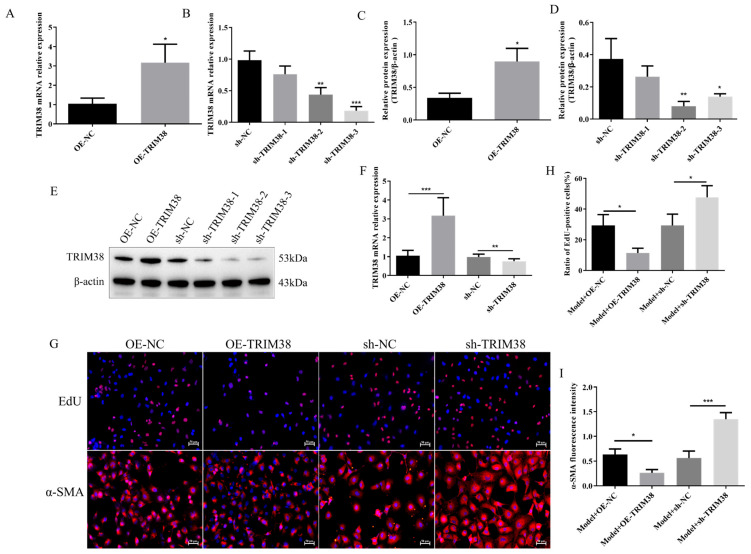
Effects of TRIM38 over-expression and knockdown on LX-2 cell proliferation and activation. (**A**,**B**) RT-qPCR validation of TRIM38 over-expression and knockdown efficiency. (**C**,**D**) Quantitative analysis of TRIM38 protein. (**E**) Representative Western blot image. (**F**) TRIM38 mRNA expression in stably transfected cell lines. (**G**) Representative immunofluorescence images. (**H**) EdU assay for cell proliferation. (**I**) Immunofluorescence detection of α-SMA expression. *n* = 3–4, Scale bar: 50 μm. Groups: Blank, Control (TGF-β1), and α-hederin (TGF-β1 + α-hederin). * *p* < 0.05, ** *p* < 0.01, and *** *p* < 0.001.

To directly validate TRIM38 function, this study established stably transfected LX-2 cell lines over-expressing (OE-TRIM38) and knocking down (sh-TRIM38) TRIM38. First, efficient transfection and regulatory effects were confirmed via RT-qPCR and Western blot analysis (Figure 4A–F). Compared to the OE-NC group, both mRNA and protein expression levels of TRIM38 were significantly elevated in the OE-TRIM38 group. Conversely, TRIM38 expression was successfully suppressed in the sh-TRIM38 group relative to the sh-NC group. Furthermore, EdU cell proliferation assays (Figure 4G,H) demonstrated that TRIM38 over-expression significantly inhibited LX-2 cell proliferation, whereas TRIM38 knockdown markedly promoted cell proliferation. Immunofluorescence detection of the cellular activation marker α-SMA further demonstrated (Figure 4G,I) that under TGF-β1-induced activation, TRIM38 over-expression significantly reduced α-SMA fluorescence intensity, whereas TRIM38 knockdown further enhanced its expression. These data directly prove that TRIM38 itself possesses inhibitory effects on hepatic stellate cell proliferation and activation.

**Figure 5:**
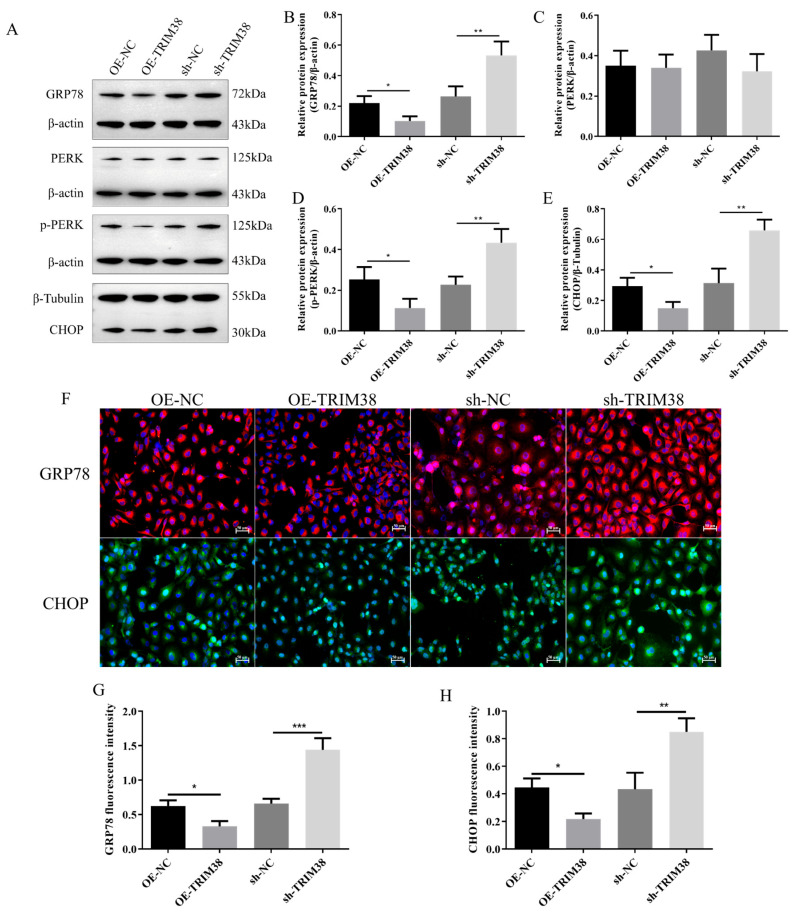
Effects of TRIM38 over-expression and knockdown on TGF-β1 induced endoplasmic reticulum stress in LX-2 cells. (**A**) Representative Western blot image. (**B**–**E**) Quantitative analysis of GRP78, PERK, p-PERK, and CHOP quantification. (**F**) Representative immunofluorescence images. (**G**,**H**) Immunofluorescence detection of GRP78 and CHOP. *n* = 3–4. Scale bar: 50 μm. Groups: Blank, Control (TGF-β1), and α-hederin (TGF-β1 + α-hederin). * *p* < 0.05, ** *p* < 0.01, and *** *p* < 0.001.

To further investigate the molecular mechanisms underlying TRIM38 function, this study examined its effects on key proteins in the ERS pathway. Western blot analysis (Figure 5A–E) revealed that in the TRIM38 over-expression group, the expression levels of ERS marker proteins GRP78, CHOP, and the activated form p-PERK were significantly reduced compared to the OE-NC group. Conversely, TRIM38 knockdown produced the opposite effect, significantly up-regulating the expression of these ERS-associated proteins. Immunofluorescence experiments provided visual confirmation (Figure 5F–H): compared to the OE-NC group, GRP78 and CHOP fluorescence intensity were significantly reduced in the OE-TRIM38 group. Conversely, fluorescence signals for both proteins were markedly enhanced in the sh-TRIM38 group. These results indicate that TRIM38 negatively regulates TGF-β1-induced endoplasmic reticulum stress in LX-2 cells.

### 3.4. α-Hederin Inhibits LX-2 Cell Activation and Alleviates Endoplasmic Reticulum Stress by Up-Regulating TRIM38

**Figure 6:**
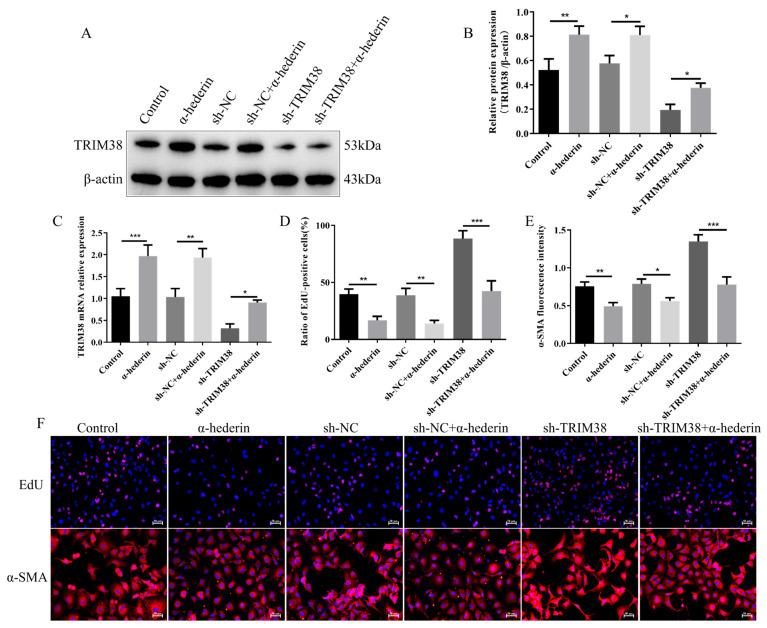
α-Hederin inhibits LX-2 cell proliferation and activation by upregulating TRIM38. (**A**) Representative Western blot image. (**B**) Western blot detection of TRIM38 protein expression. (**C**) RT-qPCR detection of TRIM38 mRNA expression. (**D**) EdU assay for cell proliferation. (**E**) Immunofluorescence detection of α-SMA. (**F**) Representative immunofluorescence images. *n* = 3–4. Scale bar: 50 μm. Groups: Blank, Control (TGF-β1), and α-hederin (TGF-β1 + α-hederin). * *p* < 0.05, ** *p* < 0.01, and *** *p* < 0.001.

To determine whether α-hederin functions depend on TRIM38, we validated the effects of α-hederin in a TRIM38-knockdown LX-2 cell model. As shown in Figure 6A–C, compared to the group with TRIM38 knockdown alone (sh-TRIM38), co-treatment with α-hederin (sh-TRIM38 + α-hederin) partially reversed the reduction in TRIM38 at both mRNA and protein levels, indicating that α-hederin positively regulates TRIM38 expression at the transcriptional level. At the cellular functional level, EdU experiments revealed (Figure 6D,F) that α-hederin treatment significantly reduced cell proliferation in the TRIM38-knockdown background. Concurrently, immunofluorescence detection of the cell activation marker α-SMA (Figure 6E,F) showed that α-hederin treatment also significantly attenuated α-SMA fluorescence intensity compared to the knockdown-only group. However, compared to the Control without TRIM38 knockdown, the inhibitory effects of α-hederin on proliferation and activation were markedly attenuated in TRIM38-deficient cells. These results indicate that although α-hederin can partially up-regulate TRIM38 and exert some inhibitory effects on proliferation and activation, its full protective function largely depends on the adequate expression of TRIM38.

**Figure 7:**
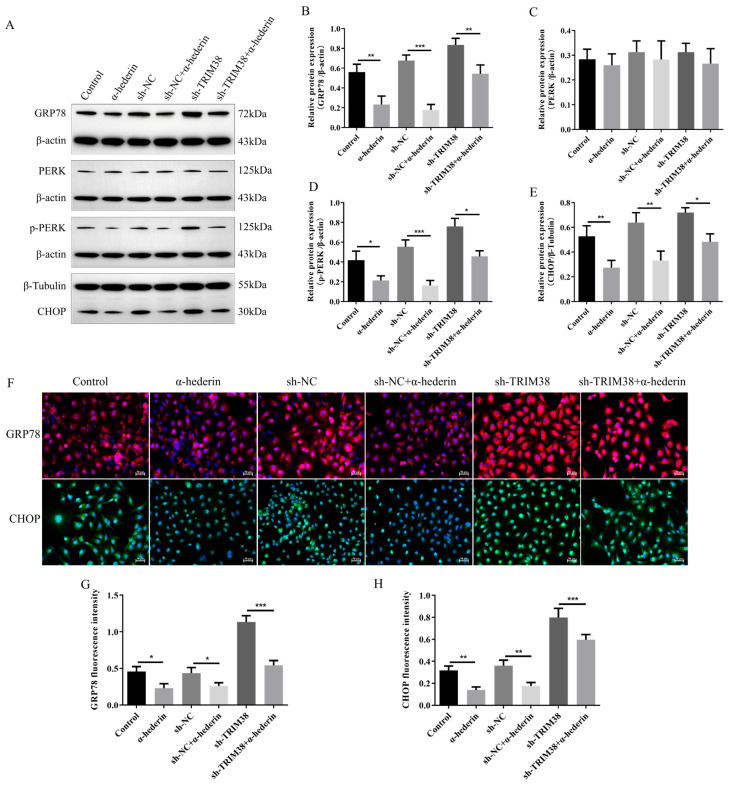
α-Hederin alleviates TGF-β1-induced ERS in LX-2 cells by up-regulating TRIM38. (**A**) Representative Western blot image. (**B**–**E**) quantitative analysis of GRP78, PERK, p-PERK, and CHOP proteins. (**F**) Representative immunofluorescence images. (**G**,**H**) Immunofluorescence detection of GRP78 and CHOP. *n* = 3–4. Scale bar: 50 μm. Groups: Blank, Control (TGF-β1), and α-hederin (TGF-β1 + α-hederin). * *p* < 0.05, ** *p* < 0.01, and *** *p* < 0.001.

Further mechanistic investigations revealed that TRIM38 is also a key molecule essential for α-hederin to alleviate endoplasmic reticulum stress. Western blot analysis (Figure 7A–E) demonstrated that in TRIM38-knockdown LX-2 cells (sh-TRIM38), α-hederin intervention still significantly down-regulated ERS marker proteins GRP78, CHOP, and p-PERK, while total PERK protein levels remained unchanged. This trend was corroborated by immunofluorescence analysis (Figure 7F–H): compared to the sh-TRIM38-only group (sh-TRIM38), the combined treatment group (sh-TRIM38 + α-hederin) exhibited significantly reduced fluorescence intensity for GRP78 and CHOP. Notably, however, α-hederin exhibited markedly reduced efficacy in suppressing ERS in TRIM38-deficient cells compared to the Control without TRIM38 knockdown. These findings indicate that while α-hederin retains some ERS-alleviating effects under low TRIM38 expression, its full functional potential is highly dependent on intact TRIM38 expression. Combining the findings from Figure 6 and Figure 7, this study confirms that α-hederin primarily suppresses endoplasmic reticulum stress by up-regulating TRIM38 expression, thereby exerting its inhibitory effect on hepatic stellate cell activation.

### 3.5. α-Hederin Alleviates CCl_4_-Induced Liver Dysfunction and Pathological Changes in Mice

**Figure 8:**
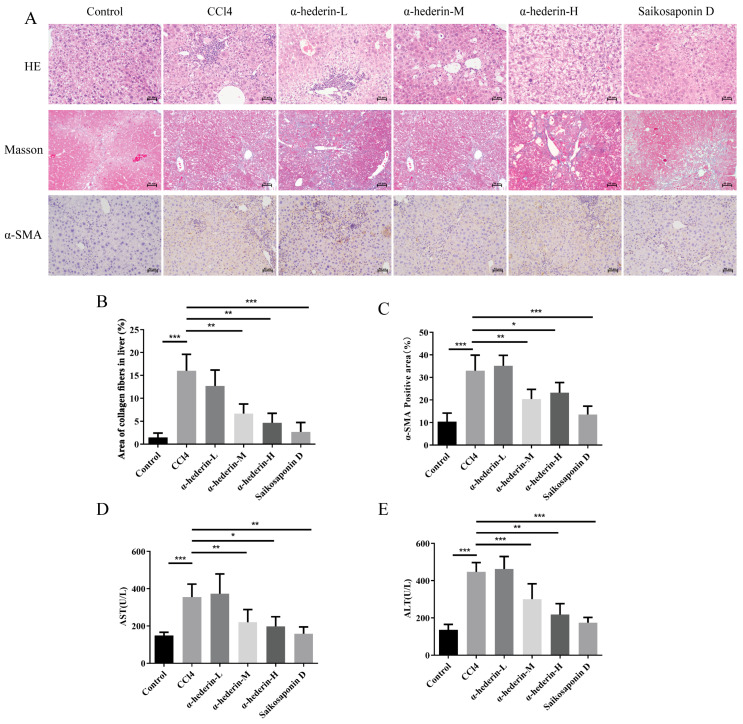
α-Hederin ameliorates CCl4-induced liver function injury and pathological changes in mice. (**A**) Representative images of HE staining, Masson staining, and α-SMA immunohistochemistry (Scale bar: 50 μm). (**B**) Quantitative analysis of collagen area percentage in Masson staining. (**C**) Quantitative analysis of α-SMA-positive area. (**D**,**E**) Serum levels of ALT and AST. *n* = 3–4. Scale bar: 50 μm. * *p* < 0.05, ** *p* < 0.01, and *** *p* < 0.001.

HE staining revealed (Figure 8A) that the CCl_4_ group exhibited extensive inflammatory cell infiltration and widespread hepatocyte degeneration and necrosis, whereas intervention with a moderate dose of α-hederin significantly alleviated the degree of inflammation and necrosis, with the hepatic lobule structure tending to remain intact, demonstrating a clear hepatoprotective effect. In contrast, the high-dose group exhibited structural abnormalities such as hepatocyte vacuolisation, suggesting that excessively high doses may induce a certain degree of toxicity. Masson’s staining results (Figure 8A,B) revealed extensive deposition of blue collagen fibers within the liver tissue of the CCl_4_ group, forming distinct fibrous septa. In the α-hederin-M group, the area of collagen deposition was significantly reduced compared with the model group, and the degree of fibrosis was markedly alleviated; however, the effects in both the low- and high-dose groups were unsatisfactory. The results of α-SMA immunohistochemical staining (Figure 8A,C) showed that the area and intensity of α-SMA-positive staining in the liver tissue of the CCl_4_ group were significantly increased, suggesting extensive activation of HSCs; In the α-hederin-M group, α-SMA expression was markedly lower than in the model group, with a reduction in positive areas and weaker staining, indicating that HSC activation was effectively suppressed; however, the down-regulation of α-SMA in both the low- and high-dose groups was less effective than in the medium-dose group. Serological assays (Figure 8D,E) further confirmed that serum ALT and AST levels were significantly elevated in the CCl_4_ group, whereas intervention with the medium dose of α-hederin resulted in a marked reduction in ALT and AST levels compared with the model group. These findings are consistent with the pathological and functional indicators, indicating that an appropriate dose of α-hederin exerts significant anti-fibrotic and hepatoprotective effects in vivo, with the medium dose (α-hederin-M) representing the optimal intervention concentration.

### 3.6. α-Hederin Upregulates TRIM38 In Vivo to Suppress Endoplasmic Reticulum Stress

**Figure 9:**
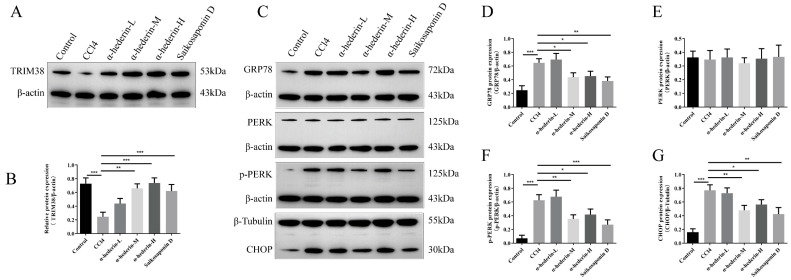
α-Hederin inhibits endoplasmic reticulum stress in vivo by upregulating TRIM38. (**A**,**B**) Western blot detection of TRIM38 protein expression in liver tissue and quantitative analysis. (**C**–**G**) Western blot detection of GRP78, PERK, p-PERK, and CHOP protein expression and quantitative analysis. *n* = 3–4. * *p* < 0.05, ** *p* < 0.01, and *** *p* < 0.001.

To verify whether the in vivo mechanism of action is consistent with that observed in cellular experiments, this study examined the expression of TRIM38 and the levels of endoplasmic reticulum stress-related proteins in liver tissue. Western blot results (Figure 9) showed that, compared with the Control group, TRIM38 protein expression was significantly down-regulated in the liver tissue of the CCl_4_ model group, whilst the expression of ERS marker proteins GRP78, CHOP, and p-PERK was substantially increased, suggesting that the development of liver fibrosis is accompanied by down-regulation of TRIM38 and strong activation of the ERS.

Following α-hederin intervention, TRIM38 protein expression in the α-hederin-M group recovered significantly compared with the model group. In close agreement with the results of the cellular experiments, as TRIM38 expression was up-regulated, the protein levels of ERS-related markers GRP78, CHOP, and p-PERK were consequently significantly down-regulated, whilst total PERK protein remained stable.

## 4. Discussion

Liver fibrosis is a relatively common liver disease clinically. It exhibits a degree of reversibility in its early stages. Without timely treatment, the progression of the disease can lead to cirrhosis or even hepatocellular carcinoma [32]. Many patients are diagnosed only when the fibrosis has already reached a severe stage. The exact causes of liver fibrosis remain incompletely understood, as it results from the interaction of multiple genes and factors [33,34]. It is now widely recognized that collagen deposition, over-expression of fibronectin, and the release of immune-related inflammatory factors are the primary drivers of liver fibrosis [35,36,37]. Recent studies further reveal that ERS plays a pivotal role in the fibrotic process. Persistent ERS activates the unfolded protein response (UPR), promoting HSC activation and extracellular matrix synthesis, forming a synergistic pro-fibrotic network with signals like TGF-β1 [12,38]. TGF-β1 is currently recognized as the most potent inducer of liver fibrosis, capable of converting quiescent HSCs into an activated phenotype, leading to substantial ECM deposition [39]. In this study, we selected 10 ng/mL TGF-β1 to treat LX-2 cells for 24 h, successfully establishing an in vitro liver fibrosis cell model for subsequent mechanism exploration.

In recent years, research on treatments for liver fibrosis has shifted from single-target approaches toward multi-target synergistic strategies. Natural products have emerged as a hotspot in anti-fibrotic drug development due to their structural diversity, high biological activity, and low toxicity [40,41]. α-Hederin is a characteristic pentacyclic triterpenoid saponin with a monosaccharide chain, found in various plants including Pulsatilla chinensis, Hedera helix L., Nigella sativa L., and Lens culinaris Medik. It exhibits antiviral, anti-inflammatory, and antitumor activities [26]. Previous studies suggest potential value of α-hederin in endoplasmic reticulum stress and liver fibrosis [25,27]. This study first reveals the mechanism by which α-hederin alleviates endoplasmic reticulum stress through up-regulating the expression of E3 ubiquitin ligase TRIM38, thereby inhibiting hepatic stellate cell activation and proliferation. This discovery not only provides a novel molecular explanation for the anti-fibrotic activity of α-hederin but also suggests that the TRIM38-endoplasmic reticulum stress axis may represent a potential new target for liver fibrosis intervention.

This study first evaluated the toxicity of α-hederin in LX-2 cells, confirming its safety at low concentrations. Based on this, cellular experiments demonstrated that α-hederin significantly suppressed the expression of α-SMA, a marker of TGF-β1-induced hepatic stellate cell activation, and effectively reduced cellular proliferation capacity, preliminarily suggesting its anti-fibrotic effects. Further transcriptomic analysis revealed that α-hederin significantly up-regulates TRIM38 expression, with DEGs enriched in endoplasmic reticulum stress-related pathways, providing crucial clues for elucidating its mechanism of action.

ERS represents a critical adaptive response to excessive protein folding loads. However, under sustained stimulation, excessive ERS promotes inflammation, apoptosis, and fibrosis through its downstream signaling pathways [12,38]. This study focused on the PERK-CHOP axis, one of the classic ERS pathways, and found that the over-expression of both α-hederin and TRIM38 significantly suppressed the activation of this pathway (as evidenced by down-regulation of GRP78, p-PERK, and CHOP). It should be noted that endoplasmic reticulum stress also involves two other crucial pathways: IRE1α/XBP1s and ATF6, both of which play key roles in regulating cell survival, metabolism, and stress responses. This study chose to focus on the PERK-CHOP pathway primarily based on the following three considerations: (1) The PERK-related pathway is a classic UPR pathway. During endoplasmic reticulum stress, PERK is released and activated by GRP78, subsequently catalyzing eIF2α to induce its phosphorylation, thereby inhibiting translation initiation and protein synthesis while promoting CHOP transcription and expression [42]; (2) During the early phase of TGF-β1 stimulation, PERK phosphorylation levels rapidly increase, subsequently inducing ATF4 and CHOP expression. Compared to IRE1α cleavage or ATF6 transport, PERK exhibits a more sensitive response to HSC activation, explaining why intervention at this node can most rapidly block downstream collagen transcription [43,44]; (3) Studies confirm CHOP directly binds the COL1A1 promoter to enhance its transcription, while IRE1-XBP1s primarily regulates metabolic enzymes. Blocking PERK-CHOP more precisely suppresses the HSC secretory phenotype while preserving hepatocyte adaptive functions, reducing potential toxicity [45,46]. This study aims to preliminarily elucidate the core pathway, which proceeds from α-hederin to TRIM38 and ultimately suppresses HSC activation through endoplasmic reticulum stress inhibition. Given that the PERK-CHOP branch is most directly linked to fibrotic output, it was selected as the mechanistic focal point. This does not negate the roles of IRE1 or ATF6 pathways in liver fibrosis; rather, they may exert specific regulatory functions in different etiologies, cell types, or disease stages. In subsequent studies, we will further examine IRE1 splicing activity, XBP1s nuclear translocation, and ATF6 activation to comprehensively map the overall regulatory landscape of α-hederin and TRIM38 on the ERS network.

TRIM38 is a key member of the TRIM (triple motif) protein family and functions as an E3 ubiquitin ligase, which catalyzes the ubiquitination of substrates and participates in the post-translational modification of proteins. Its structure comprises a typical RING domain, two B-box domains, a curled-coil domain, and a C-terminal PRY-SPRY domain, collectively supporting its roles in diverse cellular processes [21]. Previous studies suggest that TRIM38 exerts protective effects in fibrosis and related metabolic diseases. For instance, TRIM38 mitigates cardiac fibrosis following myocardial infarction by inhibiting TAK1 activation [47]; and in the context of non-alcoholic fatty liver disease, TRIM38 promotes TAB2 degradation, thereby inhibiting the TAK1–MAPK signaling cascade and alleviating hepatic lipid accumulation, inflammation, and fibrosis progression [20]. These findings collectively suggest that TRIM38 plays a crucial role in suppressing tissue fibrosis and metabolic disorders, although its specific role in HSC activation and ERS regulation remains unclear.

Functional gain-of-function and loss-of-function experiments in this study directly confirm the core role of TRIM38. Over-expression of TRIM38 mimics the effects of α-hederin, namely suppressing ERS and HSC activation; conversely, knocking down TRIM38 exacerbates ERS and promotes HSC activation. More importantly, rescue experiments revealed that upon TRIM38 knockdown, the ability of α-hederin to mitigate ERS, as well as its associated inhibition of HSC activation and proliferation, was significantly attenuated. These findings strongly support that TRIM38 functions as a key downstream effector molecule mediating the actions of α-hederin. Consistent with the existing literature, TRIM38 exhibits anti-fibrotic and anti-inflammatory effects in both myocardial fibrosis and NAFLD, acting through mechanisms involving inhibition of inflammatory pathways such as TAK1/NF-κB [19,20]. This study extends its function to regulating ERS homeostasis within HSCs, revealing a novel dimension of TRIM38′s role in liver fibrosis. We hypothesize that TRIM38 may exert its effects through its E3 ubiquitin ligase activity, ubiquitinating key components of the ERS pathway (such as GRP78 and PERK) to modulate their stability or activity, thereby attenuating the intensity of the ERS response. This hypothesis warrants further validation through immunoprecipitation and ubiquitination assays in subsequent studies. Co-IP experiments have confirmed an endogenous interaction between TRIM38 and GRP78. Given the E3 ubiquitin ligase properties of TRIM38, we hypothesize that it may regulate the stability or activity of GRP78 through ubiquitination, thereby influencing the extent of PERK pathway activation and ultimately attenuating the intensity of the endoplasmic reticulum stress response. This hypothesis requires further validation through subsequent ubiquitination experiments to determine the specific type of modification TRIM38 imposes on GRP78 and its functional consequences.

Animal experiments further validated the findings observed at the cellular level. In a CCl_4_-induced mouse model of liver fibrosis, a moderate dose of α-hederin (4 mg/kg) significantly reduced serum ALT and AST levels and improved histopathological damage to the liver; Haematoxylin and Eosin (HE) and Masson’s staining confirmed that it reduced inflammatory infiltration, hepatocyte necrosis, and collagen deposition. Mechanistically, α-hederin significantly upregulated TRIM38 expression in liver tissue whilst downregulating ERS marker proteins GRP78, p-PERK, and CHOP, consistent with the results of the cellular experiments. Notably, high-dose α-hederin (8 mg/kg) did not demonstrate superior efficacy and even led to hepatocyte vacuolisation, suggesting the potential for dose-dependent toxicity; the medium dose is therefore the optimal choice.

Of course, this study also has certain limitations. Firstly, all mechanistic experiments were conducted in the LX-2 cell line; although this cell line retains key characteristics of activated HSCs, it still differs from primary HSCs in vivo in terms of genetic background and microenvironmental responsiveness. Further validation using isolated and cultured primary HSCs is required in the future. This study confirms that TRIM38 interacts with GRP78; however, further investigation is required to elucidate precisely how TRIM38, as an E3 ubiquitin ligase, regulates the functional state of GRP78 (such as the type of ubiquitination, protein stability, or subcellular localisation). Furthermore, the specific upstream mechanism by which α-hederin upregulates TRIM38 remains to be elucidated. We hypothesize that this may occur via the following pathways: (1) as a natural small molecule, α-hederin may directly bind to the TRIM38 promoter region or associated transcription factors (such as NF-κB, AP-1, etc.), thereby enhancing TRIM38 transcriptional activity; (2) by indirectly upregulating its expression through the inhibition of TRIM38′s negative regulators or miRNAs. The aforementioned hypotheses require further validation through experiments such as dual luciferase reporter assays, ChIP-seq, and molecular docking.

In summary, this study has elucidated a novel mechanism by which α-hederin combats liver fibrosis at the cellular and animal levels by upregulating TRIM38 and inhibiting endoplasmic reticulum stress mediated by the PERK pathway. These findings add a new link to the molecular network underlying liver fibrosis and provide important experimental evidence for targeting TRIM38 in the treatment of liver fibrosis, as well as for the development of α-hederin or its derivatives as anti-fibrotic candidate drugs. Future studies should validate these findings in primary HSCs and various animal models, elucidate the precise molecular mechanisms by which TRIM38 regulates ERS, and investigate the effects of α-hederin on other ERS pathways, thereby laying a solid foundation for the development of precision anti-fibrotic therapies based on ERS regulation.

## 5. Conclusions

A novel mechanism by which α-hederin suppresses GRP78-PERK-CHOP-mediated endoplasmic reticulum stress through the upregulation of TRIM38, thereby inhibiting hepatic stellate cell activation and liver fibrosis, not only deepens our understanding of the anti-fibrotic effects of natural products but also provides a potential new target for the targeted treatment of liver fibrosis.

## Figures and Tables

**Table 1:** Sequences of the RT-qPCR primers used for each gene.

Gene	Forward Primer (5′→3′)	Reverse Primer (5′→3′)
*TRIM38*	TGGGCTGTGAAGCTGGAAAC	TGGTTACTGCATAAGGCCCC
*β-actin*	ATTTTTGTACACACAATGC	TGACCGGTGTCCCTGACGAT

## Data Availability

The raw data supporting the conclusions of this article will be made available by the authors on request.

## Supplementary Materials

The following supporting information can be downloaded at: https://www.mdpi.com/article/10.3390/biomedicines14040829/s1, Table S1: DEGs; Table S2: GSEA.

## Abbreviations

The following abbreviations are used in this manuscript:

HSCs	Hepatic stellate cells
ERS	Endoplasmic reticulum stress
DEGs	Differentially expressed genes
TRIM38	Tripartite motif-containing protein 38

## References

[1] He Z., Yang D., Fan X., Zhang M., Li Y., Gu X., Yang M. (2020). The Roles and Mechanisms of lncRNAs in Liver Fibrosis. Int. J. Mol. Sci..

[2] Delgado M.E., Cárdenas B.I., Farran N., Fernandez M. (2021). Metabolic Reprogramming of Liver Fibrosis. Cells.

[3] Campos-Murguía A., Ruiz-Margáin A., González-Regueiro J.A., Macías-Rodríguez R.U. (2020). Clinical assessment and management of liver fibrosis in non-alcoholic fatty liver disease. World J. Gastroenterol..

[4] Du X., Niu R., Liu X., Wu F., Yang X., Ma X., Zhang J., Zhou H., Shao L., Wang S. (2025). Nanomedicines in the Treatment of Liver Fibrosis: A Review. Int. J. Nanomed..

[5] Guo Y.C., Lu L.G. (2020). Antihepatic Fibrosis Drugs in Clinical Trials. J Clin. Transl. Hepatol..

[6] Cerrito L., Galasso L., Iaccarino J., Pizzi A., Termite F., Esposto G., Borriello R., Ainora M.E., Gasbarrini A., Zocco M.A. (2025). Present and Future Perspectives in the Treatment of Liver Fibrosis. Pharmaceuticals.

[7] Liu X.Y., Zhang W., Ma B.F., Sun M.M., Shang Q.H. (2024). Advances in Research on the Effectiveness and Mechanism of Active Ingredients from Traditional Chinese Medicine in Regulating Hepatic Stellate Cells Autophagy Against Hepatic Fibrosis. Drug Des. Dev. Ther..

[8] Lee C., Kim M., Han J., Yoon M., Jung Y. (2021). Mesenchymal Stem Cells Influence Activation of Hepatic Stellate Cells, and Constitute a Promising Therapy for Liver Fibrosis. Biomedicines.

[9] Roh Y.J., Kim H., Choi D.W. (2025). Metabolic Sparks in the Liver: Metabolic and Epigenetic Reprogramming in Hepatic Stellate Cells Activation and Its Implications for Human Metabolic Diseases. Diabetes Metab. J..

[10] Merens V., Knetemann E., Gürbüz E., De Smet V., Messaoudi N., Reynaert H., Verhulst S., van Grunsven L.A. (2025). Hepatic stellate cell single cell atlas reveals a highly similar activation process across liver disease aetiologies. JHEP Rep..

[11] Trivedi P., Wang S., Friedman S.L. (2021). The Power of Plasticity-Metabolic Regulation of Hepatic stellate cells. Cell Metab..

[12] Hanquier Z., Misra J., Baxter R., Maiers J.L. (2023). Stress and Liver Fibrogenesis: Understanding the Role and Regulation of Stress Response Pathways in Hepatic Stellate Cells. Am. J. Pathol..

[13] Maiers J.L., Malhi H. (2019). Endoplasmic Reticulum Stress in Metabolic Liver Diseases and Hepatic Fibrosis. Semin. Liver Dis..

[14] Saaoud F., Lu Y., Xu K., Shao Y., Praticò D., Vazquez-Padron R.I., Wang H., Yang X. (2024). Protein-rich foods, sea foods, and gut microbiota amplify immune responses in chronic diseases and cancers—Targeting PERK as a novel therapeutic strategy for chronic inflammatory diseases, neurodegenerative disorders, and cancer. Pharmacol. Ther..

[15] Huang Z., Zhou L., Liu B., Li X., Sang Y. (2025). Endoplasmic reticulum stress aggravates ferroptosis via PERK/ATF4/HSPA5 pathway in UUO-induced renal fibrosis. Front. Pharmacol..

[16] Liu R., Zhu M., Chen J., Gai J., Huang J., Zhou Y., Wan Y., Tu C. (2023). Identification and Characterization of a Novel Nanobody Against Human CTGF to Reveal Its Antifibrotic Effect in an in vitro Model of Liver Fibrosis. Int. J. Nanomed..

[17] Zhang Y., Tan X., Wang L., Ji D., Zhang C., Peng W., Zhu R., Wang X., Zhou J., Feng Y. (2025). TRIM38 Suppresses the Progression of Colorectal Cancer via Enhancing CCT6A Ubiquitination to Inhibit the MYC Pathway. Adv. Sci..

[18] Li Q., Wu H., Huang Y., Yekefenhazi D., Zou W., Han F. (2025). Characterization and Functional Analysis of Trim38 in the Immune Response of the Large Yellow Croaker (*Larimichthys crocea*) Against *Pseudomonas plecoglossicida* Infection. Int. J. Mol. Sci..

[19] Lu Z., Deng M., Ma G., Chen L. (2022). TRIM38 protects H9c2 cells from hypoxia/reoxygenation injury via the TRAF6/TAK1/NF-κB signalling pathway. PeerJ.

[20] Yao X., Dong R., Hu S., Liu Z., Cui J., Hu F., Cheng X., Wang X., Ma T., Tian S. (2023). Tripartite motif 38 alleviates the pathological process of NAFLD-NASH by promoting TAB2 degradation. J. Lipid Res..

[21] Pang Y., Wu L., Xia J., Xu X., Gao C., Hou L., Jiang L. (2025). Trim38 attenuates pressure overload-induced cardiac hypertrophy by suppressing the TAK1/JNK/P38 signaling pathway. Int. J. Mol. Med..

[22] Zhang J., Zhang Y., Ren Z., Yan D., Li G. (2023). The role of TRIM family in metabolic associated fatty liver disease. Front. Endocrinol..

[23] Zeng J., Zhao G. (2023). α-Hederin regulates macrophage polarization to relieve sepsis-induced lung and liver injuries in mice. Open Med..

[24] Belmehdi O., Taha D., Abrini J., Ming L.C., Khalid A., Abdalla A.N., Algarni A.S., Hermansyah A., Bouyahya A. (2023). Anticancer properties and mechanism insights of α-hederin. Biomed. Pharmacother..

[25] Wang Q., Feng H., Li Z., Wu Q., Li L., Sun D., Tan J., Fan M., Yu C., Xu C. (2023). α-Hederin induces human colorectal cancer cells apoptosis through disturbing protein homeostasis. Chem. Biol. Interact..

[26] Meng D., Ren M., Li M., Wang M., Geng W., Shang Q. (2024). Molecular mechanism of α-Hederin in tumor progression. Biomed. Pharmacother..

[27] Chang Y., Gao X., Jiang Y., Wang J., Liu L., Yan J., Huang G., Yang H. (2024). Alpha-hederin reprograms multi-miRNAs activity and overcome small extracellular vesicles-mediated paclitaxel resistance in NSCLC. Front. Pharmacol..

[28] Yuan Y., Liu X., Zhou T., Zhou Z., Gong M., Li Y. (2025). *Polygonatum sibiricum* polysaccharide alleviates liver fibrosis through the TGF-β/Smad signaling pathway and reduces collagen. Mol. Med. Rep..

[29] Ni L., Yang L., Lin Y. (2024). Recent progress of endoplasmic reticulum stress in the mechanism of atherosclerosis. Front. Cardiovasc. Med..

[30] Zhang Y., Han L., Wang Y., Wang M. (2025). The Role of the PI3K/Akt/mTOR Pathway in Atherosclerosis: Mechanisms, Therapeutic Potential, and Emerging Targeted Treatments. Curr. Atheroscler. Rep..

[31] Chen X., Shi C., He M., Xiong S., Xia X. (2023). Endoplasmic reticulum stress: Molecular mechanism and therapeutic targets. Signal Transduct. Target. Ther..

[32] Ravndal L., Lindvig K.P., Jensen E.L., Sunde A., Nassehi D., Thiele M., Krag A., Kjosavik S. (2023). Algorithms for early detection of silent liver fibrosis in the primary care setting—A scoping review. Expert Rev. Gastroenterol. Hepatol..

[33] Ratziu V., Boursier J. (2024). Confirmatory biomarker diagnostic studies are not needed when transitioning from NAFLD to MASLD. J. Hepatol..

[34] Liu F., Tang C.S.M., Chung P.H.Y. (2024). A narrative review of genes associated with liver fibrosis in biliary atresia. Transl. Pediatr..

[35] Kozlov D.S., Rodimova S., Filatov P., Mozherov A., Timashev P.S., Zyuzin M.V., Kuznetsova D.S. (2025). Genomic medicine in hepatology: Mechanisms and liver treatment strategies. Mol. Med..

[36] Feng L., Chen X., Huang Y., Zhang X., Zheng S., Xie N. (2023). Immunometabolism changes in fibrosis: From mechanisms to therapeutic strategies. Front. Pharmacol..

[37] Graham J., Raghunath M., Vogel V. (2019). Fibrillar fibronectin plays a key role as nucleator of collagen I polymerization during macromolecular crowding-enhanced matrix assembly. Biomater. Sci..

[38] Xia M., Li J., Martinez Aguilar L.M., Wang J., Trillos Almanza M.C., Li Y., Buist-Homan M., Moshage H. (2025). Arctigenin Attenuates Hepatic stellate cell Activation via Endoplasmic Reticulum-Associated Degradation (ERAD)-Mediated Restoration of Lipid Homeostasis. J. Agric. Food Chem..

[39] Dewidar B., Meyer C., Dooley S., Meindl-Beinker A.N. (2019). TGF-β in Hepatic stellate cell Activation and Liver Fibrogenesis-Updated 2019. Cells.

[40] Wang T., Lu Z., Sun G.F., He K.Y., Chen Z.P., Qu X.H., Han X.J. (2024). Natural Products in Liver Fibrosis Management: A Five-Year Review. Curr. Med. Chem..

[41] Qian H., Tao X., Yuan L., Wang X., Yu B. (2025). Editorial: Applications of medicinal plants and their metabolites in fibrotic disease: Novel strategies, mechanisms, and their impact on clinical practice. Front. Pharmacol..

[42] Ibrahim I.M., Abdelmalek D.H., Elfiky A.A. (2019). GRP78: A cell’s response to stress. Life Sci..

[43] Morita M., Tokumoto Y., Watanabe T., Imai Y., Yukimoto A., Shimamoto T., Yano R., Okazaki Y., Nakamura Y., Yoshida O. (2025). Endoplasmic reticulum stress sensor protein PERK in hepatic stellate cells promotes the progression of hepatocellular carcinoma via p38δ MAPK/IL-1β axis. Sci. Rep..

[44] Koo J.H., Lee H.J., Kim W., Kim S.G. (2016). Endoplasmic Reticulum Stress in Hepatic stellate cells Promotes Liver Fibrosis via PERK-Mediated Degradation of HNRNPA1 and Up-regulation of SMAD2. Gastroenterology.

[45] Wang L., Jin X.S., Dong H.J., Ou G.M., Lai X.Y., Zhuang H., Li T., Xiang K.H. (2023). Establishment of a reporter system for estimating activation of human hepatic stellate cells based on COL1A1 promoter and enhanced green fluorescent protein. Beijing Da Xue Xue Bao Yi Xue Ban.

[46] Chen K., Wang Y., Yang J., Klöting N., Liu C., Dai J., Jin S., Chen L., Liu S., Liu Y. (2024). EMC10 modulates hepatic ER stress and steatosis in an isoform-specific manner. J. Hepatol..

[47] Lu Z., Hao C., Qian H., Zhao Y., Bo X., Yao Y., Ma G., Chen L. (2022). Tripartite motif 38 attenuates cardiac fibrosis after myocardial infarction by suppressing TAK1 activation via TAB2/3 degradation. iScience.

